# Supplementary material for: The hypoxic response extends lifespan through a bioaminergic and peptidergic neural circuit
Source: eLife. 2026 Nov 6;14:RP107651. doi: 10.7554/eLife.107651 (PMC13743077; doi:10.7554/eLife.107651)
Supplement: Peer review history

# Editor report: eLife Assessment

**DOI:** 10.7554/eLife.107651.3.sa0

**Authors:** Sylvia Lee

**Affiliations:** Cornell University United States

**Keywords:** Convincing, Fundamental

This **fundamental** study, supported by **convincing** evidence, identifies the specific neuronal components that enable HIF-1 signaling in specific serotonergic neurons to regulate effectors in peripheral tissues. Specifically, stabilizing HIF-1 in ADF and NSM serotonergic neurons is sufficient to extend lifespan and healthspan in *C. elegans*, and these physiological benefits require several oxygen-sensing neurons, as well as neurotransmitters (GABA and tyramine), and a neuropeptide (NLP-17). The strengths of the study include rigorous experimental design, high quality data, the use of multiple genetic tools, and functional dissection using the well-validated *C. elegans* model.

# Referee report: Reviewer #1 (Public review)

**DOI:** 10.7554/eLife.107651.3.sa1

[Editors' note: this version has been assessed by the Reviewing Editor without further input from the original reviewers. The authors have addressed the comments raised in the previous round of review.]

Summary:

In this study by Kitto et al., the authors set out to identify specific signaling components regulating the hypoxic response from the neurons to the periphery and which components are required for lifespan extension. Their previous work had shown that expression of a stabilized HIF-1 mutant in the nervous system extends lifespan through the serotonin receptor SER-7 and leads to the induction of fmo-2 in the intestine. In the current study, they mapped the precise neural circuits required for this response, as well as the signaling mediators. Their work reveals that neurotransmitters GABA and tyramine, and the neuropeptide NLP-17, act downstream of neuronal HIF-1 to convey a "hypoxic signal" to peripheral tissues. Through cell-type-specific expression studies, targeted knockouts, and comprehensive lifespan analysis, the authors provide robust evidence to support their conclusions. The insights gained from the study are both moving the field forward as they advance our understanding of neuro-peripheral hypoxic signaling, but they also lay the groundwork for potential therapeutic strategies aimed at the modulation of such signaling pathways.

Strengths:

(1) This study provides new evidence further delineating signaling components required for hypoxic signaling-mediated longevity, from the nervous system to the periphery. Using a rigorous approach where they express stabilized HIF-1 mutant selectively in ADF, NSM, and HSN serotonergic neurons, followed by cell-type-specific tph-1 knockouts to pinpoint ADF-dependent serotonin signaling as essential for both lifespan extension and intestinal fmo-2 induction.

This was followed by generating 11 transgenic lines that drive SER-7 expression under distinct neuron-specific promoters, to systematically tease out in which of 27 candidate neurons SER-7 functions to mediate hypoxia-induced longevity. This ultimately highlighted the RIS interneuron as the required signaling hub.

(2) As the intestine lacks direct neuronal innervation, the authors employ neuron-specific RNAi (TU3311 strain) and dense core vesicle analyses to identify that the neuropeptide NLP-17 is required to transmit the hypoxic signal from RIS to induce fmo-2 in the intestine.

(3) Overall, the paper is very well written. The experiments were carried out carefully and thoroughly, and the conclusions drawn are also well supported by the results they are showing.

# Referee report: Reviewer #2 (Public review)

**DOI:** 10.7554/eLife.107651.3.sa2

Summary:

The authors aimed to identify the specific neurons, neurotransmitters, and neuropeptides that mediate the longevity effects of the hypoxic response in *C. elegans*. By genetically dissecting the pathway downstream of HIF-1, they define a neural circuit involving ADF serotonergic neurons, the SER-7 receptor in the RIS interneuron, tyraminergic signaling from RIM, and neuropeptide NLP-17, ultimately linking neuronal hypoxic sensing to pro-longevity signaling in the intestine.

Strengths:

The study employs a diverse genetic toolkit, including neuron-specific transgenes, tissue-specific knockouts and rescues, RNAi knockdowns, allowing the authors to pinpoint causality, sufficiency, and necessity with high resolution. The comprehensive mapping of cell-nonautonomous signaling adds depth to our understanding of how HIF and serotonin signaling interface with aging pathways. The conclusions are supported by consistent survival assays and fmo-2 gene expression analyses.

# Referee report: Reviewer #3 (Public review)

**DOI:** 10.7554/eLife.107651.3.sa3

Summary:

This study found that ADF serotonergic neurons have a significant role in extending lifespan mediated by HIF-1, as well as serotonin receptor SER-7 in the GABAergic RIS interneurons. The author focuses on the sufficiency and necessity of components from the central nervous system and how they contribute to aging upon hypoxia.

Previous work from the lab has identified that the stabilization of HIF-1 in neurons is sufficient to extend lifespan through the serotonin receptor, SER-7, which subsequently activates fmo-2 in the intestine and leads to lifespan extension. Building on this, the author sought to determine which serotonergic neurons are involved and found that serotonin signaling in ADF neurons is required for lifespan extension mediated by HIF-1.

The author next tested which subset of neurons requires Ser-7 expression to rescue hypoxic response. They found that ser-7 expression in multiple neurons is sufficient to induce fmo-2, with the top candidate being the RIS neuron. Ablation of the RIS neuron did not extend lifespan, suggesting that ser-7 expression in the RIS neuron is required for lifespan extension, positioning it as a key component in the longevity signaling pathway.

The author also investigated neurotransmitters and found that GABA and tyramine are important components in this circuit. They showed that the tyramine receptor called tyra-3 is required for vhl-1-mediated longevity. Given that tyra-3 is expressed in oxygen- and carbon dioxide-sensing neurons, the author demonstrated that these sensing neurons work downstream of serotonin signaling. Lastly, the author screened neuropeptide/receptor binding pairs and identified NLP-17 as playing a role in hypoxia-mediated longevity.

Originality and Significance:

This research is significant in that it uncovers components that are sufficient and necessary for lifespan extension via the hypoxic response. It provides comprehensive data supporting longevity induced by HIF-1-mediated hypoxic response, in conjunction with fmo-2, a longevity gene, as demonstrated in previous work from the lab. Moreover, it provides a number of new transgenic worm tools for *C. elegans* and aging communities.

Conclusions:

This study provides insights into how hypoxic response regulates aging in a cell non-autonomous manner, outlining a potential circuit involving neurons, neurotransmitters, and neuropeptides.

# Author comment: Author response

**DOI:** 10.7554/eLife.107651.3.sa4

**Authors:** Elizabeth S Kitto, Shijiao Huang, Mira Bhandari, Cassie Tian, Rebecca L Cox, Safa Beydoun, Emily Wang, Danielle Shave, Hillary Miller, Sarah A Easow, Ella Henry, Eugene Chung, Megan L Schaller, Scott F Leiser

**Affiliations:** University of Michigan Ann Arbor United States; Kansas State University Manhattan United States; University of Michigan Ann Arbor United States; University of Michigan Ann Arbor United States; University of Michigan Ann Arbor United States; University of Michigan Ann Arbor United States; University of Michigan Ann Arbor United States; University of Michigan Ann Arbor United States; University of Michigan Ann Arbor United States; University of Michigan Ann Arbor United States; University of Michigan Ann Arbor United States; University of Michigan Ann Arbor United States; University of Michigan Ann Arbor United States; University of Michigan Ann Arbor United States

The following is the authors’ response to the original reviews.

We thank the reviewers and editors for their time and valuable input into improving our manuscript. The reviewers recognized the value of this work while identifying places where further explanation and/or additional experiments could strengthen the manuscript. We greatly appreciate this feedback and have addressed reviewer comments through additional experiments and necessary textual edits.

In response to the reviewer comments, our principal data-driven changes to revise the manuscript include: (1) testing how stabilizing HIF-1 in the ADF and NSM neurons affects healthspan; (2) measuring for interactions between HIF-1 stabilization in serotonergic neurons and the mt-UPR; and (3) measuring *nlp-17* expression downstream of HIF-1 stabilization in serotonergic neurons. We also made textual changes including: (1) clarifying that our study focuses specifically on the *vhl-1*-mediated genetic hypoxic response; (2) summarizing which parts of the working model are experimentally validated vs. speculative; and (3) expanding our discussion of future directions needed to understand the epistasis of the many signals acting in this circuit. Our responses to each reviewer comment are below.

**Public Reviews:**

**Reviewer #1 (Public review):**

Summary:

In this study by Kitto et al., the authors set out to identify specific signaling components regulating the hypoxic response from the neurons to the periphery and which components are required for lifespan extension. Their previous work had shown that expression of a stabilized HIF-1 mutant in the nervous system extends lifespan through the serotonin receptor SER-7 and leads to the induction of fmo-2 in the intestine. In the current study, they mapped the precise neural circuits required for this response, as well as the signaling mediators. Their work reveals that neurotransmitters GABA and tyramine, and the neuropeptide NLP-17, act downstream of neuronal HIF-1 to convey a "hypoxic signal" to peripheral tissues. Through cell-type-specific expression studies, targeted knockouts, and comprehensive lifespan analysis, the authors provide robust evidence to support their conclusions. The insights gained from the study are both moving the field forward as they advance our understanding of neuro-peripheral hypoxic signaling, but they also lay the groundwork for potential therapeutic strategies aimed at the modulation of such signaling pathways.

We appreciate the reviewer’s positive assessment of the topic and general interest in this work.

Strengths:

(1) This study provides new evidence further delineating signaling components required for hypoxic signaling-mediated longevity, from the nervous system to the periphery. Using a rigorous approach where they express stabilized HIF-1 mutant selectively in ADF, NSM, and HSN serotonergic neurons, followed by cell-type-specific tph-1 knockouts to pinpoint ADF-dependent serotonin signaling as essential for both lifespan extension and intestinal fmo-2 induction.

This was followed by generating 11 transgenic lines that drive SER-7 expression under distinct neuron-specific promoters, to systematically tease out in which of 27 candidate neurons SER-7 functions to mediate hypoxia-induced longevity. This ultimately highlighted the RIS interneuron as the required signaling hub.

(2) As the intestine lacks direct neuronal innervation, the authors employ neuron-specific RNAi (TU3311 strain) and dense core vesicle analyses to identify that the neuropeptide NLP-17 is required to transmit the hypoxic signal from RIS to induce fmo-2 in the intestine.

(3) Overall, the paper is very well written. The experiments were carried out carefully and thoroughly, and the conclusions drawn are also well supported by the results they are showing.

Weaknesses:

Overall, I don't see many weaknesses. One point relates to their read-outs, which rely heavily on lifespan measurements and fmo-2 induction without evaluating other physiological processes that serotonin or NLP-17 might affect. For translational relevance, it would be valuable to assess or mention potential adverse effects, such as changes in reproduction, pharyngeal pumping, or proteostasis capacity (proteostasis capacity specifically in the tissue showing fmo-2 upregulation).

We thank the reviewer for the positive review and fully agree and acknowledge that the primary readouts used in this work, *fmo-2* induction and lifespan, may not reflect other important elements of health and physiology. To address this weakness, we have performed three measurements of healthspan (pumping, thrashing, and maximum velocity), in the ADF and NSM HIF-1 stabilized strains at young adulthood and at middle age. We focused on examining these HIF-1 stabilized strains rather than our *nlp-17* knockout animals because stabilizing HIF-1 in the NSM or ADF neurons is sufficient to extend lifespan. *nlp-17* is necessary, but it remains unclear whether *nlp-17* signaling is sufficient to extend lifespan. Our new data show that both ADF- and NSM-specific HIF-1 stabilization had no effect on pumping rate in young (day 1 of adulthood) worms. In aged animals (day 12 of adulthood), however, NSM-, but not ADF-, specific HIF-1 stabilization rescued the pumping rate decline in *hif-1* knockout compared to WT worms (new Fig. S1C). Similarly in the new thrashing data, NSM-, but not ADF-, specific HIF-1 stabilization rescued the thrashing rate decline in the *hif-1* knockout young and aged worms (new Fig. S1D). Lastly, our new data show that ADF and NSM HIF-1 stabilization had no effect on average or maximum movement speed at days 1 and 5 of adulthood (new Fig. S1E-F). Together, these results indicate that genetic activation of the hypoxic response in NSM neurons but not in the ADF neurons could improve healthspan.

While we did not examine reproductive capacity in these strains, we have expanded our discussion section to mention the importance of fully characterizing other elements of physiology, health, and behavior in future work.

“Another limitation of this work is that it uses lifespan as the main readout for organismal health. While genetic manipulations that extend lifespan often improve stress resistance and healthspan [8,80], longevity manipulations can also have adverse effects on reproduction [81,82] and behavior [83,84]. In this study, we find that HIF-1 stabilization in the ADF neurons does not prevent the deleterious effects of *hif-1* knockout on mobility, but that HIF-1 stabilization in the NSM neurons may attenuate age-related decline in pumping and thrashing (Fig. S1). However, future work should examine whether other modifications to this pathway, such as manipulations to RIM, RIS, or NLP-17 signaling, influence healthspan in addition to lifespan. It will be important for future studies to determine whether various components of this pathway affect both longevity and the response to different types of stressors like oxidative stress, proteotoxic stress, and infection, as HIF-1 activity also interacts with multiple stress responses [41,42,39,40,43].”

While lifespan assays and fmo-2 expression do provide strong evidence, incorporating additional markers of stress resistance could strengthen the link between hypoxic signaling and organismal health as well.

We also measured *hsp-6* expression via qPCR in the serotonergic neuron-specific HIF-1 stabilized strains to determine whether these conditions that lead to upregulated *fmo-2* may also affect the mt-UPR. Interestingly, we find that stabilizing HIF-1 in either the ADF or NSM serotonergic neurons decreases *hsp-6* expression relative to WT worms (new Fig. S1G). This could suggest either that the mt-UPR response is impaired in these worms, or that HIF-1 stabilization decreases proteotoxic stress leading to a lower basal level of *hsp-6*. Although this method of measurement did not allow us to interrogate whether these changes occur in the specific tissues where *fmo-2* is upregulated, we have expanded our discussion to emphasize that further investigation of cell- and tissue-specificity within the hypoxic response should be a focus of future work.

Finally, we agree that it is important to examine whether activating the hypoxic response promotes stress resistance in addition to longevity. We did not focus on this element of the hypoxic response in this work because HIF activity is known to promote adaptive stress-responses to some stressors like infection [7,8] and oxidative stress [9,10], while simultaneously impairing the proteosasis stress response [11]. We have added this important information to our introduction section, and have expanded our discussion section to emphasize that a key future direction will be to test which specific components of this pathway also facilitate stress resistance.

Introduction Section Modification:

“However, the physiological changes induced by the hypoxic response are broad and involve adaptations such as increased vascularization, metabolic rewiring, and changes in cell survival pathways. In mammals, some of these same adaptations can be detrimental, as mutations in components of the hypoxic response have been linked to conditions like cancer and cardiovascular disease [12-15]. Additionally, HIF activity is essential to promote some forms of stress resistance to infection [9,10] and oxidative stressors [7,8] but can have detrimental effects on proteostasis [11].”

Discussion Section Modification:

“Another limitation of this work is that it uses lifespan as the main readout for organismal health. While genetic manipulations that extend lifespan often improve stress resistance and healthspan [1,2], longevity manipulations can also have adverse effects on reproduction [3,4] and behavior [5,6]. In this study, we find that HIF-1 stabilization in the ADF or NSM neurons has little effect on mobility in young and aged animals. However, future work should examine whether other modifications to this pathway, such as manipulations to RIM, RIS, or NLP-17 signaling, influence healthspan in addition to lifespan. It will be important for future studies to determine whether various components of this pathway affect both longevity and the response to different types of stressors like oxidative stress, proteotoxic stress, and infection, as HIF-1 activity also interacts with multiple stress responses [7,8,9-11].”

**Reviewer #2 (Public review):**

Summary:

The authors aimed to identify the specific neurons, neurotransmitters, and neuropeptides that mediate the longevity effects of the hypoxic response in *C. elegans*. By genetically dissecting the pathway downstream of HIF-1, they define a neural circuit involving ADF serotonergic neurons, the SER-7 receptor in the RIS interneuron, tyraminergic signaling from RIM, and neuropeptide NLP-17, ultimately linking neuronal hypoxic sensing to pro-longevity signaling in the intestine.

Strengths:

The study employs a diverse genetic toolkit, including neuron-specific transgenes, tissue-specific knockouts and rescues, RNAi knockdowns, allowing the authors to pinpoint causality, sufficiency, and necessity with high resolution. The comprehensive mapping of cell-nonautonomous signaling adds depth to our understanding of how HIF and serotonin signaling interface with aging pathways. The conclusions are supported by consistent survival assays and fmo-2 gene expression analyses.

Weaknesses:

A key limitation is the lack of clear evidence showing epistasis of so many identified molecular/neuronal components downstream of HIF-1 and serotonin. Thus, the mechanisms of how a diverse set of molecules/neurons coordinate and mediate neuronal HIF-1 effects on intestinal fmo-2 and longevity remain murky.

We thank the reviewer for these important points. We agree that the epistatic relationships between ADF serotonin, RIM tyramine, RIS GABA, and neuropeptide NLP-17 signaling remain unclear within this pathway. Determining the epistatic relationships of each signal within this complex pathway will require: (1) generating genetic manipulations to each identified signaling component that may mimic *vhl-1* knockout to promote longevity; (2) crossing these new strains into multiple genetic knockouts we identified as required for *vhl-1* mediated longevity; and (3) measuring the lifespans of each double and triple mutant. We are very interested in testing the epistatic relationships between all of these molecules and neurons, and believe this extensive follow-up exploration will generate significant future results.

To better address these limitations of the current work, we have added a summary table to Fig. 7 as well as a paragraph to our discussion section. This table and paragraph better explain which components of our working model have been tested for necessity, sufficiency, and epistasis, and which components of this model remain unclear (Fig. 7). See updated Fig. 7 with added table.

Updated discussion section detailing this limitation:

“While many individual neurosignaling components are essential for genetic activation of the hypoxic response to extend lifespan, their epistasis is unclear (Fig. 7B). Most components of the pathway identified in this work act downstream of *vhl-1* depletion, and upstream of *fmo-2* induction (summarized in Fig. 7A-B). However, the order of each signal between these two endpoints is only predicted based on *C. elegans* neural wiring and the overlap between various identified signals and cells. For example, we hypothesize in our working model that GABA may be produced by the RIS neuron in this circuit because RIS is the primary GABAergic neuron required for *vhl-1*-mediated longevity. Alternatively, it is possible that GABA is produced by a different cell that either acts in series or in parallel with RIS signaling. In order to determine the order of each signaling component, future studies should generate genetic manipulations to each signaling component that may mimic *vhl-1* knockout to promote longevity, cross these new strains into knockouts of other signals required for *vhl-1* mediated longevity; and measure the lifespans of each double and triple mutant. This approach would also narrow down which signals are downstream of the genetic activation of the hypoxic response, and which are sufficient to extend lifespan upstream of the hypoxic response in a normoxic environment. One notable target for further exploration is the SER-7 expressing RIS neuron, which plays a role in sleep [16] and stress resistance [17], and can extend lifespan when optogenetically activated under normoxic conditions [18].”

Some rescue strategies may inadvertently cause non-physiological expression.

This is a great point. We bring attention to this limitation in the discussion section. Our cell-specific knockouts (ADF *tph-1* KO, Fig. 1E) or ablations (RIS ablation, Fig. 2C) data showed that the neurons identified using rescue strains (i.e., *tph-1* in the ADF and *ser-7* in RIS) are likely not false positives. However, we did not generate a RIM-specific knockout or ablation strain to confirm our *tdc-1* results and have added this limitation to the discussion of these results.

Discussion of rescue strategy limitations and the need for a RIM-specific knockout:

“The circuit-mapping approaches employed in this work are also impacted by limitations in cell-specific genetic modifications and in the use of RNAi knockdown. For example, cell-specific rescue constructs can sometimes lead to unintended rescues in other cell types due to cell-nonautonomous signaling. Because all serotonin-producing neurons also express the serotonin reuptake transporter *mod-5*, serotonin produced by one cell in our rescue strains could be taken up by other serotonin-producing neurons, leading to unintended signaling effects. This may also be true of the uv1 and RIM tyraminergic rescue strains, although little is known about tyramine reuptake in *C. elegans*. Two cells identified via tissue-specific rescue experiments (ADF and RIS, Fig. 1G and Fig. 2D) were also found to be necessary via cell-specific knockout (ADF *tph-1* KO, Fig. 1E; RIS ablation, Fig. 2C), decreasing the likelihood of a false positive from the rescue strain technique. However, the role of the RIM neuron was identified via a *tdc-1* rescue strain and was not validated using a RIM-specific knockout (Fig. 7B). Therefore, the contribution of RIM signaling to this circuit is less well-validated, and a RIM-specific *tdc-1* knockout strain should be examined in future work.”

Additionally, environmental hypoxia was not tested in parallel, so the claim on "hypoxia response" throughout the manuscript is not justified by genetic manipulation alone, and the translational relevance of the genetic manipulations remains somewhat uncertain.

We thank the reviewer for identifying the need for additional specificity in our language. We agree that it is critical to make it clear that this paper only examines genetic mimics of hypoxia, rather than environmental hypoxia, and have replaced every occurrence of “the hypoxic response” with either (1) “genetic activation of the hypoxic response”, or (2) describing the specific manipulation used in that experiment (e.g., “*vhl-1* mediated longevity”). We also agree that examining the similarities and differences between genetic and environmental activation is a key next step towards evaluating the translational potential of this pathway. We discuss this in Discussion and are excited to interrogate these differences in future work.

“While this study identifies many neural signals required for *vhl-1* knockdown or knockout to extend lifespan, one key limitation of this work is the potential differences between genetic and environmental methods of inducing the hypoxic response. While *vhl-1* knockdown or knockout leads to HIF-1 stabilization by blocking its proteasomal degradation, it also results in hydroxylated HIF-1. This contrasts with environmental hypoxia, in which HIF-1 remains stable because it cannot be hydroxylated. While HIF-1 is stabilized and localized to the nucleus in both cases, there are differences in transcriptional outcomes between stable hydroxylated and unhydroxylated states [19,20]. Additionally, we did not explore the effects of alternative genetic activators of the hypoxic response such as HIF-1 hydroxylase PHD/EGL mutants, which may provide further insight into how different manipulations of the hypoxic response impact longevity. Future work should interrogate the similarities and differences between the circuits driving longevity in response to environmental hypoxia, *vhl-1* knockdown, HIF-1 stabilization, and PHD/EGL knockdown.”

**Reviewer #3 (Public review):**

Summary:

This study found that ADF serotonergic neurons have a significant role in extending lifespan mediated by HIF-1, as well as serotonin receptor SER-7 in the GABAergic RIS interneurons. The author focuses on the sufficiency and necessity of components from the central nervous system and how they contribute to aging upon hypoxia.

Previous work from the lab has identified that the stabilization of HIF-1 in neurons is sufficient to extend lifespan through the serotonin receptor, SER-7, which subsequently activates fmo-2 in the intestine and leads to lifespan extension. Building on this, the author sought to determine which serotonergic neurons are involved and found that serotonin signaling in ADF neurons is required for lifespan extension mediated by HIF-1.

The author next tested which subset of neurons requires Ser-7 expression to rescue hypoxic response. They found that ser-7 expression in multiple neurons is sufficient to induce fmo-2, with the top candidate being the RIS neuron. Ablation of the RIS neuron did not extend lifespan, suggesting that ser-7 expression in the RIS neuron is required for lifespan extension, positioning it as a key component in the longevity signaling pathway.

The author also investigated neurotransmitters and found that GABA and tyramine are important components in this circuit. They showed that the tyramine receptor called tyra-3 is required for vhl-1-mediated longevity. Given that tyra-3 is expressed in oxygen- and carbon dioxide-sensing neurons, the author demonstrated that these sensing neurons work downstream of serotonin signaling. Lastly, the author screened neuropeptide/receptor binding pairs and identified NLP-17 as playing a role in hypoxia-mediated longevity.

Originality and Significance:

This research is significant in that it uncovers components that are sufficient and necessary for lifespan extension via the hypoxic response. It provides comprehensive data supporting longevity induced by HIF-1-mediated hypoxic response, in conjunction with fmo-2, a longevity gene, as demonstrated in previous work from the lab. Moreover, it provides a number of new transgenic worm tools for *C. elegans* and aging communities.

We thank the reviewer for the positive assessment of the manuscript. We appreciate all suggestions for further improving the work and have made changes based on these suggestions (see details below).

Data and Methodology:

(1) The experiments were thoroughly conducted, especially the generations of strains using different neuron-type promoters and crossing into mutant strains to demonstrate sufficiency and necessity.

(2) Some figure legends from the text do not match what the data show. (Figure 6E, F, G).

We have made changes to the legends accordingly to make sure figures and legends are consistent.

(3) The lifespan graph legends are confusing and could use some revamping for better clarification.

We have updated the lifespan graph legends to provide the statistics in the same section as the labels, indicating which line is which condition (see updated Figure 1E). We hope this change better clarifies the lifespan graph legends.

Conclusions:

This study provides insights into how hypoxic response regulates aging in a cell non-autonomous manner, outlining a potential circuit involving neurons, neurotransmitters, and neuropeptides.

**Recommendations for the authors:**

**Reviewing Editor Comments:**

As suggested by the Reviewers 2 & 3, including environmental hypoxia will broaden the impact of the study. If not, the authors should consider clarifying their response as "genetic activation of the hypoxic response" (see Reviewer 2 below).

We thank the editor and reviewers 2 and 3 for this clarification. We agree that it is critical to make it clear that this paper only examines genetic mimics of hypoxia, rather than environmental hypoxia, and have replaced every occurrence of “the hypoxic response” with either (1) “genetic activation of the hypoxic response”, or (2) describing the specific manipulation used in that experiment (e.g., “*vhl-1* mediated longevity”). We also agree that examining the similarities and differences between genetic and environmental activation is a key next step towards evaluating the translational potential of this pathway. We have also expanded our discussion of this important future direction.

“While this study identifies many neural signals required for *vhl-1* knockdown or knockout to extend lifespan, one key limitation of this work is the potential differences between genetic and environmental methods of inducing the hypoxic response. While *vhl-1* knockdown or knockout leads to HIF-1 stabilization by blocking its proteasomal degradation, it also results in hydroxylated HIF-1. This contrasts with environmental hypoxia, in which HIF-1 remains stable because it cannot be hydroxylated. While HIF-1 is stabilized and localized to the nucleus in both cases, there are differences in transcriptional outcomes between stable hydroxylated and unhydroxylated states [19,20]. Additionally, we did not explore the effects of alternative genetic activators of the hypoxic response such as HIF-1 hydroxylase PHD/EGL mutants, which may provide further insight into how different manipulations of the hypoxic response impact longevity. Future work should interrogate the similarities and differences between the circuits driving longevity in response to environmental hypoxia, *vhl-1* knockdown, HIF-1 stabilization, and PHD/EGL knockdown.”

Suggested minor changes by Reviewer 3 should also be made to improve the readability of the paper.

We have made changes suggested by Reviewer 3 to improve the readability of the paper.

**Reviewer #2 (Recommendations for the authors):**

(1) Suggestions for additional experiments or analyses:

(a) To clarify the hierarchical relationships among the components of the identified circuit, epistasis experiments between serotonin, tyramine, NLP-17, and oxygen-sensing neurons (e.g., double mutants or sequential rescues) would strengthen the proposed model and help determine whether these signals act in parallel or downstream of each other.

We thank the reviewer for identifying this important caveat. As described in the public review response, we completely agree that understanding the epistasis of serotonin, tyramine, NLP-17, and oxygen-sensing neuron signaling within this pathway is important to fully test our working model. We hope to address these questions about epistasis and interactions between different signals in upcoming projects.

To clarify that the order of many of these signals remains speculative in our working model, we have added a table to Fig. 7, that summarizes what is known and unknown about the epistasis of these pathway components. We have also expanded our discussion of this limitation. See updated Fig. 7 with added table.

Updated discussion section detailing this limitation:

“While many individual neurosignaling components are essential for genetic activation of the hypoxic response to extend lifespan, their epistasis is unclear (Fig. 7B). Most components of the pathway identified in this work act downstream of *vhl-1* depletion, and upstream of *fmo-2* induction (summarized in Fig. 7A-B). However, the order of each signal between these two endpoints is only predicted based on *C. elegans* neural wiring and the overlap between various identified signals and cells. For example, we hypothesize in our working model that GABA may be produced by the RIS neuron in this circuit because RIS is the primary GABAergic neuron we found to be required for *vhl-1*-mediated longevity. Alternatively, it is possible that GABA is produced by a different cell that either acts in series or in parallel with RIS signaling. In order to determine the order of each signaling component, future studies should generate genetic manipulations to each signaling component that may mimic *vhl-1* knockout to promote longevity, cross these new strains into knockouts of other signals required for *vhl-1* mediated longevity; and measure the lifespans of each double and triple mutant.”

(b) Testing whether environmental hypoxia (e.g., 0.5-1% O₂ exposure) elicits similar neuronal requirements and fmo-2 induction as the genetic HIF-1 stabilization would validate that the described pathway is relevant to the actual hypoxic response and improve translational relevance.

We thank the reviewer for identifying the need for clarification. As also discussed in the public review section, we agree that it is important to emphasize that this paper exclusively examines genetic mimetics of hypoxia, rather than actual exposure to a hypoxic environment. To clarify this point, we have replaced every occurrence of “the hypoxic response” with either (1) “genetic activation of the hypoxic response”, or (2) describing the specific manipulation used in that experiment (ie “*vhl-1* mediated longevity”). We also agree that examining the similarities and differences between genetic and environmental activation is a key next step towards evaluating the translational potential of this pathway. We discuss this in discussion and are excited to interrogate these differences in future work.

“While this study identifies many neural signals required for *vhl-1* knockdown or knockout to extend lifespan, one key limitation of this work is the potential differences between genetic and environmental methods of inducing the hypoxic response. While *vhl-1* knockdown or knockout leads to HIF-1 stabilization by blocking its proteasomal degradation, it also results in hydroxylated HIF-1. This contrasts with environmental hypoxia, in which HIF-1 remains stable because it cannot be hydroxylated. While HIF-1 is stabilized and localized to the nucleus in both cases, there are differences in transcriptional outcomes between stable hydroxylated and unhydroxylated states [19,20]. Additionally, we did not explore the effects of alternative genetic activators of the hypoxic response such as HIF-1 hydroxylase PHD/EGL mutants, which may provide further insight into how different manipulations of the hypoxic response impact longevity. Future work should interrogate the similarities and differences between the circuits driving longevity in response to environmental hypoxia, *vhl-1* knockdown, HIF-1 stabilization, and PHD/EGL knockdown.”

(c) Functional readouts beyond lifespan and fmo-2 induction (e.g., neuronal activity monitoring or optogenetic modulation of key neurons) could help clarify how information flows through the circuit.

We thank the reviewer for this valuable suggestion. We are hoping to be able to implement these experimental techniques in future work. We agree that in combination with genetic epistasis analyses, direct measurements of neuronal signaling will greatly improve our understanding of the directionality and interactions between signals in this circuit. Our discussion section recommends employing these techniques in future studies.

“Finally, while the use of RNAi knockdown and genetic knockouts establishes the necessity of many signals within the *vhl-1*-mediated longevity circuit, the exact directionality of these signals remains unclear. It is possible that increased, decreased, or pulsatile changes in signaling through these bioamines and neuropeptides are required for genetic activation of the hypoxic response to extend lifespan. Work on *C. elegans* reversal behavior has also revealed an antagonistic relationship between RIM and RIS activity facilitated by both chemical (neuropeptide and tyramine) and electrical (gap junction) signaling [16,21]. This known interaction should also be interrogated in the context of how these cells may communicate following genetic induction of the hypoxic response. Future work in this area could use tools to measure or modify neuronal activity, such as calcium imaging or optogenetics, to begin answering these questions.”

(2) Recommendations for improving the writing and presentation:

(a) The manuscript is well written overall, but clarity would be improved by explicitly stating in the abstract and introduction that the study is based on genetic activation of the hypoxic response, rather than environmental hypoxia.

We appreciate this valuable suggestion and have updated the abstract and introduction to clarify that this work examines genetic activation of the hypoxic response.

Updated sentences from the abstract:

“Here, we interrogate the cell-nonautonomous signaling pathway downstream of genetic activation of the hypoxic response.”

“Together, these insights develop a circuit for how genetic induction of the hypoxic response cell-nonautonomously modulates ageing and suggests valuable targets for modulating ageing in mammals.”

Updated sentences from the introduction:

“In this study, we uncover key neural components of the longevity circuit initiated by genetic induction of the hypoxic response. Within this circuit, we identify individual cells, signals, and receptors necessary and/or sufficient to extend lifespan downstream of genetic activation of the hypoxic response. More specifically, we find serotonin signaling in the ADF serotonergic neurons is both necessary and sufficient to extend lifespan through genetic activation of the hypoxic response. This pathway signals through the serotonin receptor SER-7 in the RIS interneuron. We further demonstrate additional neurotransmitters (GABA and tyramine), and a neuropeptide (NLP-17) are critical for mediating these longevity effects. Finally, we identify that oxygen sensing neurons (URX, AQR, PQR and BAG) act downstream of neuronal HIF-1 in this circuit. Our insights into this longevity pathway provide a mechanistic understanding of how genetic activation of the hypoxic response delays aging and improves health.”

(b) In the discussion, clearly delineating which parts of the proposed pathway are firmly established versus inferred would aid interpretation.

We thank the reviewer for this idea, and have added the following text to the discussion:

“Evidence for the necessity, sufficiency, and epistatic relationships between each signal are summarized in new Fig. 7B. In brief, all signaling molecules presented in this work are necessary for *vhl-1* to extend lifespan. Rescuing ADF serotonin production, RIS *ser-7* expression, and RIM tyramine production is sufficient for *vhl-1* to extend lifespan. The sufficiency of oxygen sensing neurons BAG and URX/PQR/AQR, and the neuronal signals of GABA, NLP-17, and TYRA-3 to restore *vhl-1* mediated longevity remains unclear. All signals act downstream of *vhl-1*. ADF HIF-1 stabilization and SER-7 signaling act upstream of *fmo-2* induction, and the oxygen sensing neurons (BAG, URX/PQR/AQR) act upstream of or in parallel to ADF HIF-1 stabilization.”

(c) Adding a summary table or schematic that visually distinguishes necessity vs. sufficiency for each component (e.g., ADF, RIS, RIM, NLP-17) would make the overall model more accessible.

We thank the reviewer for this great suggestion and have added a table to new Fig. 7B that summarizes what is known about necessity for *vhl-1*, sufficiency for *vhl-1*, and sufficiency to extend lifespan independently of *vhl-1* for each signal (table included in response to Reviewer 2, comment 1a).

(3) Minor corrections and clarifications:

(a) Define or replace "hypoxic response" with "genetically induced hypoxic response" where appropriate to avoid conflating genetic manipulations with actual environmental hypoxia.

We appreciate this valuable suggestion and have replaced “the hypoxic response” and with “genetic activation of the hypoxic response” or “genetically induced hypoxic response” throughout the manuscript to clarify this point.

(b) All genes and alleles should be italicized per worm nomenclature.

We thank the reviewer for this comment and have reviewed the manuscript to italicize all gene names and alleles. In some locations, the protein is referred to instead of the gene using the conventional uppercase non-italicized format.

**Reviewer #3 (Recommendations for the authors):**

(1) Using vhl-1 RNAi as the sole approach to demonstrate hypoxic response appears somewhat limited, as vhl-1 is also involved in HIF-1 independent processes that can influence lifespan in *C. elegans*. Including additional downstream effectors of HIF-1, such as egl-9, or having HIF-1 nondegradable strain as validation could strengthen the findings.

We thank the reviewer for this valuable comment and agree that an important next step is to test whether these signals are also required for other genetic (HIF-1 stabilized, *egl-9*) and environmental activators of the hypoxic response to extend lifespan. We have worked to clarify that this paper focuses primarily on *vhl-1* mediated longevity throughout the text and have also included this limitation in our discussion section (for details, please see response to Reviewer 2 public review).

(2) Investigating how the healthspan is affected by serotonergic neuron-specific hypoxic responses would be interesting and could enhance understanding of the physiological mechanisms underlying lifespan extension.

We appreciate this suggestion, and have performed three measurements of healthspan (pumping, thrashing, and maximum velocity), in the ADF and NSM HIF-1 stabilized strains at young adulthood and at middle age. We found that both ADF- and NSM-specific HIF-1 stabilization had no effect on pumping rate in young (day 1 of adulthood) worms. In aged animals (day 12 of adulthood), however, NSM-, but not ADF-, specific HIF-1 stabilization rescued the pumping rate decline in hif-1 knockout compared to WT worms (new Fig. S1C). Similarly, NSM-, but not ADF-, specific HIF-1 stabilization rescued the thrashing rate decline in the hif-1 knockout young and aged worms (new Fig. S1D). ADF and NSM HIF-1 stabilization also had no effect on average or maximum movement speed at days 1 and 5 of adulthood (new Fig. S1E-F). Together, these results indicate that genetic activation of the hypoxic response in NSM neurons but not in the ADF neurons could improve healthspan.

(3) While the experiments were thoroughly performed, the connections between components such as NLP-17, GABA, and tyramine in regulating aging appear critical for establishing a "cell non-autonomous circuit." Additionally, how the potentially antagonistic roles of RIM and RIS neurons influence this axis could be interesting to further explore.

We thank the reviewer for identifying this important caveat. As described in the public review response to Reviewer 2, we completely agree that understanding the epistasis of serotonin, tyramine, NLP-17, and oxygen-sensing neuron signaling within this pathway is important to fully test our working model. Our current data showed that intestinal *fmo-2* is required for neuronal HIF-1 stabilization to extend lifespan, indicating information must be communicated between the nervous system and the intestine through serotonin, tyramine, NLP-17 and responsible neurons using a “cell non-autonomous circuit” [22]. However, we will continue to address questions about epistasis and interactions between different signals in upcoming projects to fully establish the circuit.

With respect to RIM and RIS, we value this suggestion and agree that there could be interesting signaling occurring between RIM and RIS in this circuit, as is observed in initiation of reversal behaviors [16,21]. We have updated the discussion section to mention this interesting antagonistic relationship between RIM and RIS signaling in the context of reversal behaviors:

“Finally, while the use of RNAi knockdown and genetic knockouts establishes the necessity of many signals within the *vhl-1*-mediated longevity circuit, the exact directionality of these signals remains unclear. It is possible that increased, decreased, or pulsatile changes in signaling through these bioamines and neuropeptides are required for genetic activation of the hypoxic response to extend lifespan. Work on *C. elegans* reversal behavior has also revealed an antagonistic relationship between RIM and RIS activity facilitated by both chemical (neuropeptide and tyramine) and electrical (gap junction) signaling [16,21]. This known interaction should also be interrogated in the context of how these cells may communicate following genetic induction of the hypoxic response. Future work in this area could use tools to measure or modify neuronal activity, such as calcium imaging or optogenetics, to begin answering these questions.”

(4) Does serotonergic neuron-specific rescue impact the mitochondrial unfolded protein response (mtUPR), given that serotonin signaling has been shown to modulate mtUPR?

We appreciate this question and suggestion. To determine whether activating the hypoxic response in serotonergic neurons modifies the mt-UPR, we measured *hsp-6* expression via qPCR in the ADF and NSM-specific HIF-1 stabilized strains. Interestingly, we find that stabilizing HIF-1 in either the ADF or NSM serotonergic neurons decreases *hsp-6* expression relative to WT worms new Fig. S1G. This could suggest either that the mt-UPR response is impaired in these worms, or that HIF-1 stabilization decreases proteotoxic stress leading to a lower basal level of *hsp-6*. Although this method of measurement did not allow us to interrogate whether these changes occur in the specific tissues where *fmo-2* is upregulated, we have expanded our discussion of these results to emphasize that further investigation of this response should be a focus of future work.

(5) To remain consistent with the flow of Figure 1, the authors should include fmo-2 expression in NSM:HIF-1S in addition to the ADF:HIF-1S (Figure 1I).

We appreciate this suggestion and have added NSM::HIF-1S data to Fig. 1I. We found that stabilizing HIF-1 in either the ADF or the NSM has a similar effect on *fmo-2* induction.

(6) It would greatly strengthen the RIS observation if the authors demonstrated that ablation of another neural subtype from their screen does not abolish lifespan extension by vhl-1 RNAi. This would be a good supplemental figure, but it is not necessary for the overall story.

We thank the reviewer for this suggestion and agree that ablating a *ser-7* expressing neuron that was not a hit from our screen would be an excellent additional control. We did find that ablating a non-*ser-7*-expressing neuron (the RIC, Fig. 3E) did not affect *vhl-1* mediated longevity, suggesting that impairing the signaling of any interneuron is not sufficient to disrupt the phenotype. In addition, ablating a *ser-7* expressing neuron other than RIS would provide much stronger support for this finding. We have suggested this approach to further validate this working model in the future directions section of our discussion.

“Finally, additional genetic controls could better support the role of RIS-specific *ser-7* expression in genetic activation of the hypoxic response. For example, a *ser-7* expressing neuron that was not a hit in our screen could also be ablated and tested for necessity in *vhl-1* mediated longevity. This experiment would test whether the ability of RIS ablation to attenuate *vhl-1*-mediated longevity is not a false positive driven by any disruption to *ser-7* expression.”

(7) For consistency with the rest of the manuscript, it would strengthen the hypothesis if modulating the expression of nlp-17 or its receptors impacted the intestinal activation of fmo-2 transcription.

This is a great point. We attempted this experiment, but were unable to achieve consistent results (see Author response image 1). This result could be due to indirect effects of the overexpression of *nlp-17* signaling modulating *fmo-2* induction in a complicated circuit, variability in expression of its receptor, or other complexities within the circuit.

**Author response image 1.**

(8) It would strengthen the manuscript to determine whether serotonergic, GABA, and/or tyramine signaling activate the expression or secretion of this nlp-17 neuropeptide.

We thank the reviewer for this great idea of experiment. To address this suggestion, we performed qPCR to measure *nlp-17* mRNA in WT, *hif-1* KO, ADF HIF-1 stabilized, and NSM HIF-1 stabilized strains. Compared to WT and *hif-1* KO controls, we observed no change in *nlp-17* expression when HIF-1 was stabilized in the ADF or NSM serotonergic neurons (new Fig. S5F). This could suggest either that *nlp-17* signaling acts in parallel to serotonergic signaling following genetic activation of the hypoxic response. Alternatively, neuronal HIF-1 stabilization may modify *nlp-17* splicing or translation without resulting in detectable differences in mRNA levels. Together, these data indicate NLP-17 signaling is required for longevity following genetic activation of the hypoxic response, although whether this peptide is synthesized or released in response to hypoxic response remains unclear.

We agree that it is also important to connect *nlp-17* expression and/or secretion to other components of this pathway. However, we believe the most effective experiment to confirm a connection between GABA and tyramine signaling and *nlp-17* in the context of hypoxia would be to measure *nlp-17* expression in strains that manipulate GABA and/or tyramine signaling in a manner that mimics *vhl-1* knockdown and extends lifespan. Because we have not yet validated hypoxic-response mimetics for these specific signals, we hope to first generate these strains and then measure their effect on *nlp-17* expression in future work. The importance of identifying manipulations to GABA and tyramine signaling that promote longevity has been added to our discussion section.

“While many individual neurosignaling components are essential for genetic activation of the hypoxic response to extend lifespan, their epistasis is unclear (Fig. 7B). Most components of the pathway identified in this work act downstream of *vhl-1*, and upstream of *fmo-2* induction (summarized in Fig. 7A-B). However, the order of each signal between these two endpoints is only predicted based on *C. elegans* neural wiring and the overlap between various identified signals and cells. For example, we hypothesize in our working model that GABA may be produced by the RIS neuron in this circuit because RIS is the primary GABAergic neuron required for *vhl-1*-mediated longevity. Alternatively, it is possible that GABA is produced by a different cell that either acts in series or in parallel with RIS signaling. In order to determine the order of each signaling component, future studies should generate genetic manipulations to each signaling component that may mimic *vhl-1* knockout to promote longevity, cross these new strains into knockouts of other signals required for *vhl-1* mediated longevity; and measure the lifespans of each double and triple mutant. This approach would also narrow down which signals are downstream of the genetic activation of the hypoxic response, and which are sufficient to extend lifespan upstream of the hypoxic response in a normoxic environment. One notable target for further exploration is the SER-7 expressing RIS neuron, which plays a role in sleep [16] and stress resistance [17], and can extend lifespan when optogenetically activated under normoxic conditions [18].”

References:

(1) Calabrese, E. J., Dhawan, G., Kapoor, R., Iavicoli, I. & Calabrese, V. What is hormesis and its relevance to healthy aging and longevity? Biogerontology 16, 693-707 (2015). https://doi.org/10.1007/s10522-015-9601-0

(2) Zhou, I. K., Pincus, Z. & Slack, J. F. Longevity and stress in *Caenorhabditis elegans*. Aging 3, 733-753 (2011). https://doi.org/10.18632/aging.100367

(3) Yuan, R., Hascup, E., Hascup, K. & Bartke, A. Relationships among Development, Growth, Body Size, Reproduction, Aging, and Longevity - Trade-Offs and Pace-Of-Life. Biochemistry (Mosc) 88, 1692-1703 (2023). https://doi.org/10.1134/S0006297923110020

(4) Mautz, B. S., Lind, M. I. & Maklakov, A. A. Dietary Restriction Improves Fitness of Aging Parents But Reduces Fitness of Their Offspring in Nematodes. J Gerontol A Biol Sci Med Sci 75, 843-848 (2020). https://doi.org/10.1093/gerona/glz276

(5) Duric, V., Clayton, S., Leong, L. M. & Yuan, L.-L. Comorbidity Factors and Brain Mechanisms Linking Chronic Stress and Systemic Illness. Neural Plasticity 2016, 1-16 (2016). https://doi.org/10.1155/2016/5460732

(6) Mariotti, A. The Effects of Chronic Stress On Health: New Insights Into the Molecular Mechanisms of Brain–Body Communication. Future Science OA 1 (2015). https://doi.org/10.4155/fso.15.21

(7) Bellier, A., Chen, C.-S., Kao, C.-Y., Cinar, H. N. & Aroian, R. V. Hypoxia and the Hypoxic Response Pathway Protect against Pore-Forming Toxins in *C. elegans*. PLoS Pathog 5, e1000689 (2009).

(8) Palazon, A., Goldrath, W. A., Nizet, V. & Johnson, S. R. HIF Transcription Factors, Inflammation, and Immunity. Immunity 41, 518-528 (2014). https://doi.org/10.1016/j.immuni.2014.09.008

(9) Vora, M. et al. The hypoxia response pathway promotes PEP carboxykinase and gluconeogenesis in *C. elegans*. Nature Communications 13 (2022). https://doi.org/10.1038/s41467-022-33849-x

(10) Nakazawa, S. M., Keith, B. & Simon, C. M. Oxygen availability and metabolic adaptations. Nature Reviews Cancer 16, 663-673 (2016). https://doi.org/10.1038/nrc.2016.84

(11) Fawcett, M. E., Hoyt, M. J., Johnson, K. J. & Miller, L. D. Hypoxia disrupts proteostasis in *Caenorhabditis elegans*. Aging Cell 14, 92-101 (2015). https://doi.org/10.1111/acel.12301

(12) Ohh, M., Taber, C. C., Ferens, F. G. & Tarade, D. Hypoxia-inducible factor underlies von Hippel-Lindau disease stigmata. Elife 11 (2022). https://doi.org/10.7554/eLife.80774

(13) Wind, J. J. & Lonser, R. R. Management of von Hippel-Lindau disease-associated CNS lesions. Expert Rev Neurother 11, 1433-1441 (2011). https://doi.org/10.1586/ern.11.124

(14) Lonser, R. R. et al. von Hippel-Lindau disease. Lancet 361, 2059-2067 (2003). https://doi.org/10.1016/S0140-6736(03)13643-4

(15) Kaelin, W. G. Molecular basis of the VHL hereditary cancer syndrome. Nat Rev Cancer 2, 673-682 (2002).

(16) Costa, S. W. et al. A GABAergic and peptidergic sleep neuron as a locomotion stop neuron with compartmentalized Ca2+ dynamics. Nature Communications 10 (2019). https://doi.org/10.1038/s41467-019-12098-5

(17) Wu, Y., Masurat, F., Preis, J. & Bringmann, H. Sleep Counteracts Aging Phenotypes to Survive Starvation-Induced Developmental Arrest in *C. elegans*. Curr Biol 28, 3610-3624.e3618 (2018). https://doi.org/10.1016/j.cub.2018.10.009

(18) Busack, I. & Bringmann, H. A sleep-active neuron can promote survival while sleep behavior is disturbed. PLOS Genetics 19, e1010665 (2023). https://doi.org/10.1371/journal.pgen.1010665

(19) Powell-Coffman, J. A. & Coffman, C. R. Apoptosis: Lack of oxygen aids cell survival. Nature 465, 554-555 (2010). https://doi.org/10.1038/465554a

(20) Kruempel, J. C. P. et al. Hypoxic response regulators RHY-1 and EGL-9/PHD promote longevity through a VHL-1-independent transcriptional response. Geroscience 42, 1621-1633 (2020). https://doi.org/10.1007/s11357-020-00194-0

(21) Bach, M., Bergs, A., Mulcahy, B., Zhen, M. & Gottschalk, A. (2023).

(22) Leiser, S. F. et al. Cell nonautonomous activation of flavin-containing monooxygenase promotes longevity and health span. Science (2015). https://doi.org/10.1126/science.aac9257
